# Design and Implementation of a Trust Information Management Platform for Social Internet of Things Environments

**DOI:** 10.3390/s19214707

**Published:** 2019-10-29

**Authors:** Tai-Won Um, Eunhee Lee, Gyu Myoung Lee, Yongik Yoon

**Affiliations:** 1 Department of Information and Communication Engineering, Chosun University, Gwangju 61452, Korea; twum@chosun.ac.kr; 2 School of IT Engineering, Sookmyung Women’s University, Seoul 04310, Korea; eunhee@iitp.kr; 3 Department of Computer Science, Liverpool John Moores University, Liverpool L3 3AF, UK; g.m.lee@ljmu.ac.uk

**Keywords:** trust, trust information management platform, trust index, Internet of Things, cyber physical system

## Abstract

As the vast amount of data in social Internet of Things (IoT) environments considering interactions between IoT and people is accumulated and processed through cloud and big data technologies, the services that utilize them are applied in various fields. The trust between IoT devices and their data is recognized as the core of IoT ecosystem creation and growth. Connection with suspicious IoT devices may pose a risk to services and system operation. Therefore, it is essential to analyze and manage trust information for devices, services, and people, as well as to provide the trust information to the other devices or users that need it. This paper presents a trust information management framework which contains a generic IoT reference model with trust capabilities to achieve the goal of converged trust information management. Additionally, a trust information management platform (TIMP) consisting of trust agents, trust information brokers, and trust information management systems has been proposed, which aims to provide trustworthy and safe interactions among people, virtual objects, and physical things. Implementing and deploying a TIMP enables a trustworthy ecosystem to be built while activating social IoT businesses by reducing transaction costs, as well as by eliminating the uncertainties in the use of social IoT services and data transactions.

## 1. Introduction

At the early stages of Internet of Things (IoT) technologies, physical sensors and devices were considered to be the main targets to be managed and controlled by IoT service operators for the purpose of providing sensing services to users. However, as IoT has evolved as a common service infrastructure, various applications and services of IoT have been emerging into markets in broad areas, e.g., smart home/building, health care, security, transportation, and so on. Recently, IoT has been stimulated by the advent of cyber physical systems (CPS) [1], where physical things are connected to one another and to cyber objects to provide intelligent services [2]. In CPS, the physical domain and the cyber domain are substantially the same, and both functional capabilities are connected and affect each other. 

In more recent years, studies on interactions between IoT and people such as cyber physical social systems (CPSS) [3] and social IoT (SIoT) [4,5] have been actively carried out. The paradigms of CPSS and SIoT have been expanded to encompass not only the physical and the cyber domains but also the social domain. The physical IoT domain perceives the dynamic physical environment and collects and delivers data by using physical things, while the cyber IoT domain computes and analyzes the data through one or more cyber objects, and information or knowledge useful for context awareness and decision making can be used by users in the social IoT domain through interactions among individuals and communities as well as physical things.

However, the introduction of newly developed technologies is always subject to uncertainty, which is likely to cause problems in terms of stability and security [6]. In particular, there is no guarantee of a certain level of control and reliability. If there is no trust between humans, the exchange of data and information between them is also meaningless because there is no confidence in each other [7]. Human-to-machine interactions have also proven to be unpredictable and unreliable, regardless of the normal functioning of the human and machine systems [8].

Direct connections between IoT devices occur in variable manners, increasing the complexity of IoT services and applications, and there is a high likelihood of potentially unknown risks due to this complex interaction. In addition, as the IoT application services spread to the real world and the interactions between IoT devices and users become frequent, increased suspicion about whether IoT devices and services operate for their original purposes without any problems and whether they are harmful to users is recognized as a major obstacle [9].

A matter of trust in data collection is also a critical issue in the physical IoT domain. IoT service quality will be significantly degraded by hacked or damaged devices, even though trust in the cyber IoT domain can be fully supported. Next, data processing trust should be guaranteed in the cyber IoT domain. Therefore, trust in IoT needs to be managed through the physical and the cyber IoT domains in a holistic manner. 

The expanded paradigm of IoT, including CPSS and SIoT, makes it difficult for users to grasp whether or not the neighboring things and services are reliable and credible. That is, collecting data from trustworthy physical things is the first step to providing trustworthy information and communication technology (ICT) services and applications, and proper virtual objects have to be chosen to gain trustworthy knowledge or meaningful information by analyzing and calculating data. However, current IoT infrastructures fundamentally cannot block economic and financial losses due to various malicious attacks, thus increasing user mistrust. In other words, the present security technology is a perimeter-based security solution and, as it can cope with a malicious attack on a contact point, there is a limit to the fundamental solution.

In this background, there are technical demands for verifying and confirming the trustworthiness of the SIoT based on the interactions between IoT devices, services, and people in the physical, cyber, and social IoT domains. Trust in IoT devices and data is a prerequisite for the spread and activation of SIoT-based industries and services such as smart homes, connected cars, and telemedicine. By analyzing and managing trust information for devices, services, and people as well as by providing the trust information to the other devices or users that need them, IoT devices and services will be more trustworthy and reliably used. However, existing papers on trust have mainly focused on the theoretical aspects of users’ trust analysis algorithms [10]. Thus, this paper aimed to present a practical system design and implementation based on the service model to analyse and provide trust information for service realization in alignment with the international standard – ITU-T Y.3052 (see Clause 2.1) [11].

In this paper, we designed a trust information management framework containing a generic IoT reference model with trust capabilities to achieve the goal of converged trust information management. We then proposed a trust information management platform (TIMP) consisting of trust agents, trust information brokers, and trust information management systems in SIoT environments. The design and implementation of TIMP enables trust-based, reliable, and stable services by verifying and providing trust information for data, devices, services, and users in emerging SIoT environments where people, objects, and services interact frequently.

As a typical example of TIMP-based services, this paper considered various sharing services (e.g., Airbnb and Uber) that temporarily connect offices, accommodations, automobiles, etc., owned by a particular person to other people. These services have recently emerged and have shown a high utilization rate. Unlike individuals using well-known hotels and car rental companies, because strangers have short-term lease of each other’s houses and automobiles in the sharing economy world, a tenant must confront the uncertainty and risk inherent in using such a lease service. Therefore, trust becomes a big obstacle in using and spreading such a service. From the point of view of owners of resources, since a lender lends their resource to a complete stranger, the lender has concerns about whether the complete stranger will use the resource appropriately and carefully according to the contracted terms. By the illustration of a use case, the paper also demonstrated a key operation and procedure of essential components for the analysis and use of trust information in emerging IoT services and applications to cope with the sharing economy.

The remainder of this paper is organized as follows. Background information on trust is provided in Section 2. A trust information management framework is described in Section 3. Section 4 proposes detailed components of the TIMP and presents a trust data analytics procedure, including the trust data processing and analytics to derive trust indexes of physical things, virtual objects, users, and services. In Section 5, we demonstrate the implementation of the proposed solutions and a use case for TIMP-based resource-sharing services. Finally, we summarize our work in Section 6.

## 2. Background

### 2.1. Definition and Attribution of Trust

In a lexical sense, trustworthiness is a concept that implies the integrity, power, ability, and assurance of a person or thing. Generally, trustworthiness is used as a measure of confidence that something will behave as expected, even though it lacks the ability to observe or control the environment in which it operates [6]. The concept of trust itself is very complex, with different meanings depending on the subject, situation, etc., and is influenced by various measurable and unmeasurable factors. There are also a number of trust attributes, but they frequently vary over a specific time period within a particular context. Thus, it is very difficult to make generalizations, regardless of personal preferences and situation.

According to previous research, trustworthiness is described by objective factors such as competence and reputation, along with some subjective factors such as status in social relations and physical attributes. Here, competence is a measure of the ability of a person to perform a given task based on his/her degree, qualifications, and experience, and reputation is formed based on the opinions of people who have previously interacted with the subject [4].

The term trust originated from the humanities and social sciences. Trust is thus a broad concept used in many fields and subject areas, but until now, there has been no generally agreed-upon definition. In the ICT domain, confusion arises in the use of this terminology because it is mixed with various interpretations and definitions such as information security, privacy, and reliability.

To build converged ICT services and a reliable information infrastructure, ITU-T (International Telecommunication Union Telecommunication Standardization Sector) Study Group 13 on future networks and cloud has been working on future trust ICT infrastructures and recently published Recommendation Y.3052 “Overview of trust provisioning in ICT infrastructures and services” [11] regarding the concept of trust, a trust relationship model, and trust evaluation with trust indicators and a trust index. According to the Y.3052, trust is defined as “the measurable belief and/or confidence which represents accumulated value from history and the expected value for the future”. Trust indicators represent fundamental criteria for evaluating trust in entities in ICT environments. Trust indicators can be categorized into two major groups: objective trust indicators and subjective trust indicators. Trust index is a comprehensive accumulation of trust indicators, which can evaluate and quantify the trust of entities.

### 2.2. Previous Studies on Trust in SIoT

In the early stages of IoT technologies, sensors and devices were considered to be passive objects to be managed and controlled. As people began to interact more and more closely with the circumambient physical things, IoT industries and academia began to pay much attention to the SIoT, which is defined as an IoT where things are capable of establishing social relationships with other objects autonomously with respect to people [4]. In the SIoT, a physical thing is capable of discovering and selecting other things in imitation of social relationships between people [5].

From the cognitive and subjective aspect of human’s mind, the trustworthiness of things is recognized as a key challenge for invigorating IoT services. L. Atzori et al. [5] proposes a subjective and an objective model for trust management of SIoT. The former is used to compute the trustworthiness of things on the basis of one’s own experience and the reputation of the thing. In the latter, the trustworthiness of things is determined by using distributed and stored information based on a peer-to-peer structure [12]. Ontology-based semantic models have also been used to analyze the trustworthiness of things. However, existing trust models have mainly focused on limited IoT capabilities for the physical domain and reasoning for the trustworthiness of IoT devices.

On the other hand, social networks and social media are growing rapidly and users can share their thoughts (e.g., Twitter), multimedia (e.g., YouTube), personal activities, information (e.g., Facebook), and documents or calendars (e.g., Google+) through a variety of services [13,14]. The social networks based on the technology of Web 2.0 have greatly enhanced the participation of users on the web by providing an environment where users can easily communicate with each other and easily share interesting contents such as photographs and video clips [15]. Such social networks typically represent various attributes of user profiles and user relationships—that is, between a person and a person, and between a person and content. Many people spend more time on social networking sites than ever before, and prefer to communicate and interact with friends through social media [16]. A social network is a social structure made up of a set of people and a set of links between people. The social network perspective provides a set of methods for analyzing the structure of entire social entities, as well as a variety of theories explaining the patterns observed in these structures [17]. There are some advantages of applying social networking technologies to the IoT [4]: (1) Trust can be defined and examined to leverage the degree of interactions among things; (2) discovery of objects and services can be executed scalably and effectively, as in human social networks; and (3) social network modeling and analysis can be re-used to address IoT-related issues.

In the SIoT, the trustworthiness of things is recognized as a key challenge, with its evaluation needed to determine whether or not neighboring things and services are reliable and credible. For example, in crowd-sourcing applications such as swarm intelligence, each object is used as the bearer of its specific service to the community [4]. To realize this scenario, objects need to make social relationships, including policy, activities, object profile, etc. According to Reference [5], relationships between objects in SIoT can be classified as follows [18]:

- “co-location” relationships, established among objects used always in the same place;
- “co-work” relationships, established whenever objects collaborate to provide a common IoT application;
- “parental” relationships, related to objects belonging to the same production batch (e.g., same manufacturer, same model);
- “co-ownership” relationships, established among heterogeneous objects which belong to the same user.

The main advantage of using these social relationships between objects is that objects can offer services to their owners by autonomously cooperating with other objects, irrespective of whether or not there are social connections between the owners of such objects.

In particular, this SIoT concept may play an important role in the deployment of services that depend on loosely coupled interactions among objects, the value of which lies in their capability to dynamically discover key information and services from unknown communities of objects. To realize this service based on SIoT, each object should be equipped with social functionalities to discover other social objects and to search for information and services via the object social network.

It is evident that the openness of social behavior introduces many weaknesses from a security point of view that have to be addressed appropriately before deploying relevant applications. However, the evaluation of an object’s trustworthiness can take advantage of the social network itself and can be performed with appropriate models for managing the trustworthiness of the other social objects which may behave maliciously.

Our previous work [19] presented a trustworthiness evaluation model called REK, comprising a triad of trustworthiness indicators: reputation (public evidence of a trustee), experience (personal expertise about the situation and the context), and knowledge (understanding of a trustee). The REK model covers multi-dimensional aspects of trustworthiness by incorporating heterogeneous information from personal experiences to global opinions [20]. By extending the REK model, Reference [21] proposed a quantifiable trustworthiness assessment model based on machine learning and Reference [22] proposed a novel trust model called experience–reputation (E-R) for evaluating trust relationships between any two mobile device users.

Based on our previous theoretical trust model, this paper has presented a framework for designing all components required to comprehensively cover the overall operations and procedure for trust information collecting, processing, and management, including analytics. The paper also focuses on the implementation and demonstration of a service platform with the trust solutions (i.e., TIMP) required for various services and applications in SIoT environments.

## 3. Trust Information Management Framework

In this section, we present a trust information management framework which contains a reference model and related capabilities within three IoT domains in order to achieve the design goal of converged trust information management.

### 3.1. Converged Trust Information Management

Trust information services can be used to verify trustworthiness in people, objects, and applications in various SIoT services. Many SIoT service providers need a trust information service for the purpose of maintaining quality and providing reliable and stable transactions for their services. In addition, individual users also require a trust information service for the purpose of prevention of leakage of personal data, prevention of fraudulent telephone calls, prevention of housing invasion, and security check of user devices, including in the IoT [23].

In order to provide trust information services, it is first necessary to collect trust-related data for users, devices, and applications including social, cyber, and physical areas of public, corporate, individual sectors according to the demand of SIoT service providers and users. After that, it is necessary to measure and analyze the trustworthiness of users, devices, and applications through modeling and reasoning for suitable trustworthiness analysis according to the demand of the SIoT services. In addition, a convenient trust information service interface based on a web application programming interface (API) must be provided so that various services and users can easily access the trust information service.

Such a solution should not be limited to a specific service or application, but should be widely used for verifying the reliability of users and devices in various IoT services and applications. To this end, the trust information management solution should minimize the dependency on services and applications, and the functions such as trust information analysis and management should be as common as possible so that they can be reused in various services.

**Figure 1:**
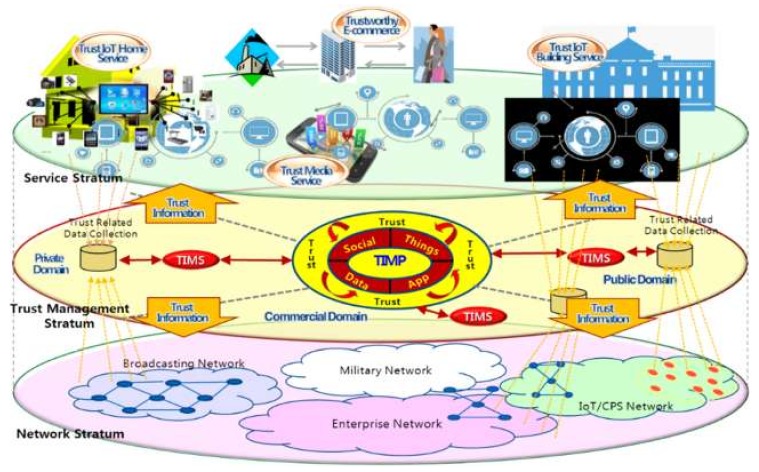
Converged trust information management.

Figure 1 shows a conceptual diagram of the converged trust information management, which consists of network stratum containing of physical devices connected to each other through a network, service stratum transferring, storing and processing data and information in various services, and trust management stratum that is responsible for analyzing and providing trust information services to SIoT service providers and users. There is a trust information management platform (TIMP) that commonly analyzes and manages trust information on the cloud. The home and building services can analyze and manage trust information within their service domains using the trust information management system (TIMS) which is dynamically allocated by the TIMP according to the software-as-a-service (SaaS) method.

A trust domain is a collection of trustworthy objects and data including users, networks, data storages and applications. To provide end-to-end trustworthy services, multiple trust domains need to be associated and the trust information maintained and managed for objects, users, and services in each trust domain should be shared with each other.

### 3.2. Generic IoT Trust Reference Model

**Figure 2:**
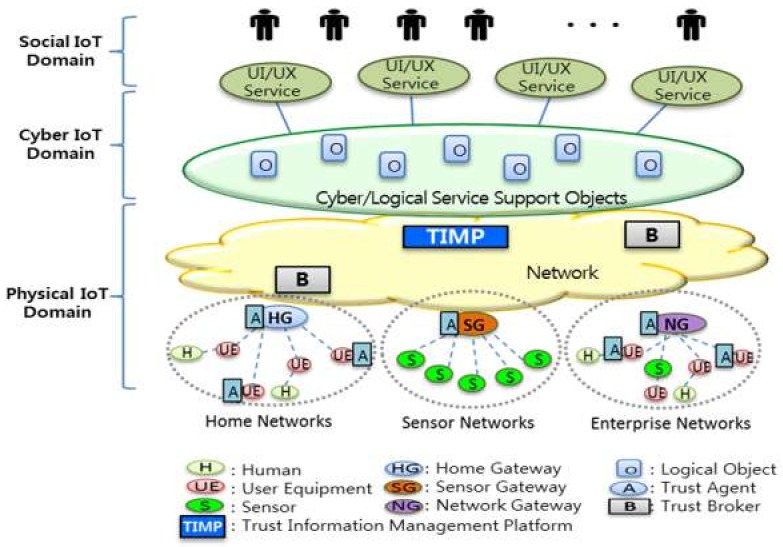
Trust in the physical, cyber, and social Internet of Things (IoT) domains.

Trust information management has been highlighted as a key issue in the mediation and handling of commercial services, as well as decision-making in business processes. Trust information management plays an important role in the IoT in the detection, monitoring, and collection of data from various kinds of devices such as sensor nodes, sensor gateways, user equipment, home gateways, and network gateways in the physical IoT domain, as well as cyber objects and services/applications in the cyber IoT domain, as shown in Figure 2. Moreover, in the social IoT domain, trust information serves as a basis for decision-making, even as people select IoT services or connect to nearby IoT devices.

Through trust information management, the collected trust data can be further aggregated, classified, and analyzed to determine an appropriate level of trust in physical things, cyber objects, and people. Moreover, it helps people to overcome perceptions of uncertainty and risk, and engages user acceptance and consumption on IoT services and applications. To provide trustworthy IoT services, all IoT entities, including applications, platforms, networks, and devices, have to properly work together through the service goal.

In general, there are three IoT domains: (1) the physical IoT domain, which perceives the dynamic physical environment and collects and delivers data; (2) the cyber IoT domain, which analyzes and processes the data from the physical IoT domain, and provides services to users; and (3) the social IoT domain, which makes decisions based on IoT data analysis or uses physical IoT devices and cyber IoT services. The physical IoT domain and the cyber IoT domain are substantially different, but both capabilities are connected and affect each other in many aspects of data, control, and management. In addition, users generate social data, information, and knowledge by themselves or through interactions among people, and cyber data and knowledge are generated through the operation of the software and processes of the cyber IoT domain. Likewise, physical data are generated from a terminal at the physical IoT domain. Trust issues such as confidentiality, integrity, and availability are important aspects of the physical, cyber, and social IoT domains that need to be considered [1].

As new services closely interact with each other in SIoT, it is necessary to analyze and manage the trust in each domain, and to analyze and manage the cross-domain trust between the other physical, cyber, or social domains. In the case of convergence among heterogeneous services in a SIoT environment, the trust information in each service must be able to be used in objects and data in other services beyond the service area. In this way, cross-service interactions require structural trust analysis and management for the service domain itself, and methods and procedures for supporting cooperation between trust-based service domains should be provided.

The growing use of IoT means the generation of large volumes of data can be expected. Collecting trustworthy data from physical things or cyber objects is the first step in providing trustworthy IoT services and applications. There are a number of different types of algorithms and systems available to extract information or knowledge from aggregated data.

A trustworthy IoT service depends on reliable cooperation between the different IoT domains, as well as each capability in the physical, cyber, and social IoT domains. In order to develop a trust analysis algorithm, the specification of trustworthy objects and attributes must precede trust modeling. Here, trust modeling involves designing a trust domain by structuring and shaping trust data in a form that enables trust inference and interpretation of behavior and state data of users, devices and services. Furthermore, corresponding trust technologies at each domain should also be described to collaborate with the IoT capabilities.

**Figure 3:**
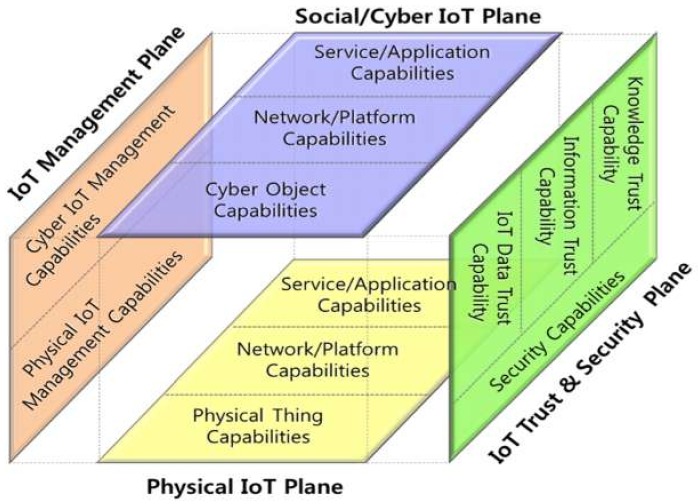
Generic IoT trust reference model and related capabilities.

Reflecting on these considerations, a reference model needs to be defined to clarify the relationship between IoT capabilities and trust capabilities, as shown in Figure 3, where the IoT trust and security plane consists of IoT data trust capabilities, information trust capabilities, knowledge trust capabilities, and security capabilities. The physical IoT plane consists of the physical IoT device, network, and platform capabilities, and the social/cyber IoT plane consists of software capabilities embedded in devices, networks, and platforms. On the other hand, the IoT management plane is responsible for the operation and management of the capabilities on the physical IoT plane and the social/cyber IoT plane.

### 3.3. Constraints on Data Acquisition

In order to analyze and provide trust information for people, objects, and applications, it is essential to collect data from public, private, corporate, and commercial areas. In the design of a trust information management framework, data related to trust should be designed in a way that reflects practical constraints such as data silos and personal data protection laws. Service providers, individuals, corporations, and government agencies maintain and manage data from economic, social, cultural, and public activities, but these data are not generally allowed to be shared and sold, because data collection may involve privacy issues for service users or device owners in most cases.

For example, user data related to media services are very useful for providing customized services and targeted advertisements. However, data collection in media services imposes serious constraints and requires trust-enabled mechanisms such as trustworthy data crawling and reasoning with policies, and some data collected by smartphones may contain sensitive information such as the location data of the owners. Because of these constraints of data collection, including users’ privacy and regulations, a data-analysis-based service basically needs a data usage and protection agreement.

In accordance with these privacy considerations, enterprises and individuals who want to use a trust service should purchase a TIMP using their own trust data, or lease the trust service in the form of a software-as-a-service (SaaS) cloud to use as a business model. Otherwise, trust information can also be obtained through a trust information broker (TIB) when trust information about people, objects, or application services held by other providers and public institutions is needed.

Trust information is required in many areas, including the commercial domain as well as the enterprise, private, and public domains. The subjects of trust information include not only people, but also various objects of social, cyber, and physical fields, such as physical objects to be traded, services on the Internet, and household appliances.

However, most data from users, devices, and services needed for trust analysis contain private data of individuals and are linked with sensitive service policies, so it is very difficult to share these data in each service domain with other services or users. In order to develop and apply a realistic TIMP to data silos, it is necessary to provide trust information in compliance with such data silos and privacy restrictions.

## 4. Trust Information Management Platform

### 4.1. TIMP Architecture

**Figure 4:**
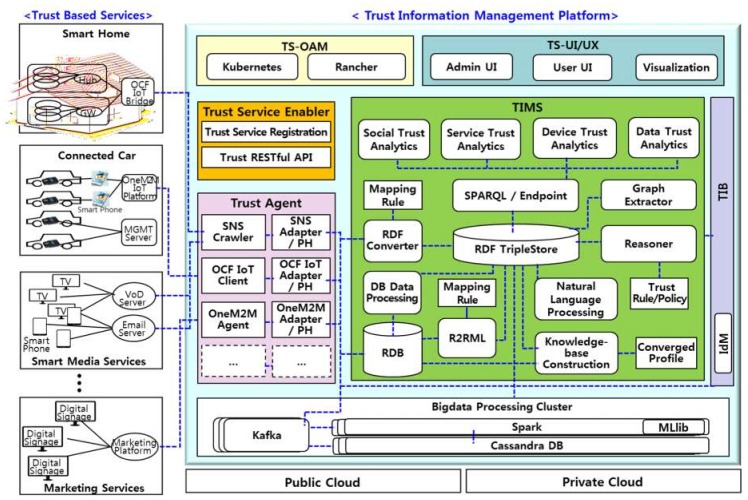
Architecture of the trust information management platform.

Considering the trust information management framework described in Section 3, this section describes the architecture of the proposed TIMP. Basically, it has been designed to create non-dependent structure for services and applications to be used in various fields. As shown in Figure 4, the TIMP consists of seven subsystems: trust service enabler (TSE), trust agent (TA), trusted information and management system (TIMS), trust information broker (TIB), trust system—operations, administration, and management (TS-OAM), trust system—user interface/user experience (TS-UI/UX), and big data processing cluster.

(1) Trust Service Enabler (TSE)

The TSE performs trust service registration from service providers and users requiring trust information, and it is responsible for dynamically generating and providing the TIMS with the following modules:

- Trust RESTful API is an interface that enables various trust system modules such as the TA, TIMS, TIB, and databases to be registered and managed in the TSE. Trust system module providers such as the TA, TIMS, TIB, and databases can receive usage fees based on their usage when their modules registered with TSE are used for trust services.
- Trust service registration performs the function of dynamically configuring and allocating a virtual TIMS to the service provider by receiving the registration of the trust information service from the service users and orchestrating the registered trust system modules using the Trust RESTful API.

(2) Trust Agent (TA)

The TA provides a number of interfaces for data collection that can collect IoT and service data from various types of IoT services, such as smart homes, connected cars, and smart media with the following modules:

- Social Networking Service (SNS) crawler periodically acquires user data from various social network services such as Facebook, Twitter, Gmail and so on.
- SNS adapter and privacy handler (PH) performs the function of anonymizing the user data received from the SNS crawler and transmitting it to the database of the TIMS. Because the TIMS stores, analyzes, and manages trust information based on anonymized personal information, it can cope with the leakage of personal information due to hacking and similar.
- OCF/OneM2M IoT clients are IoT data collection interfaces according to the OCF (Open Connectivity Foundation) standards and OneM2M standards, respectively.
- OCF/OneM2M IoT adapter and PH modules anonymize and transfer data collected from the OCF/OneM2M IoT Client to the TIMS, similar to the role described for the SNS adapter and PH.

(3) Trust Information and Management System (TIMS)

The TIMS analyzes the data of users, services, and IoT devices delivered through the TA using social network analysis techniques, machine-learning-based analysis techniques, natural language processing techniques, and ontology-based analysis techniques, and it performs functions to infer and manage the trust indexes of devices and so on with the following major modules:

- The social network analysis module serves to deduce the trust indexes among users by analyzing patterns of communication between users through social network services and e-mails. It uses ontology methods to share and deliver social network data in a systematic representation format. Several ontologies, such as friend-of-a-friend (FOAF) [24], are used to represent social networks. FOAF ontologies, which provide information extracted from user profiles and lists, are widely used to provide portability between social networking sites and to model user-generated information and content in a machine-readable manner, since they can describe their relationships and online activities. In addition, resource description framework (RDF)-based social data descriptions provide a much more effective way of representing online social networks than existing social network models. In addition, semantic web technology is also very useful for improving information retrieval performance and increasing flexibility in data access.
- A natural language processing module finds information such as stakeholder trust, IoT trust, service trust, and data trust based on text data collected from Facebook, Twitter, and Gmail, and builds a knowledge base.
- A service trust analysis module analyzes service utilization data in smart homes, connected cars, and smart media services, which are generated by the service itself.

A TIMS uses standard technologies related to the semantic web for common representation of heterogeneous IoT data collected in the physical IoT domain, and applies linked data technologies for common representation of trust information in the cyber and the social IoT domains. However, data in the social/cyber/physical domains can all be converted to RDF format, stored, and looked up and used for trust analysis. IoT and social data collected from services such as smart homes, connected cars, etc. are stored in the Non Structured Query Language (NoSQL)-based Cassandra database and SQL-based MySQL database, and are converted and delivered to the RDF-based TripleStore. By utilizing this semantic web technology, data on social networks can be integrated with data from other sources to develop more valuable data and information. Furthermore, semantic web technology is very effective in knowledge management processes that extract, maintain, and develop knowledge.

(4) Trust Information Broker (TIB)

A TIB arbitrates trust information for users, services, and IoT devices in other service domains to be received and transmitted through user consent and anonymization processing. Trust identity management (IdM) plays a role to identify whether trust objects of different service domains are the same user, because each TIMS deduces and manages trust information based on anonymization of user information.

(5) Trust System—Operations, Administration, and Management (TS-OAM)

The TS-OAM module is responsible for the operation and management of trust system modules using Kubernetes [25] and Rancher [26], which are open source projects that bring cluster management capabilities to the world of virtual machines.

In order for a TIMP to be effectively applied to various services, it is necessary for the user to easily identify user-friendly trust information for nearby IoT devices and services. For administrators, it is important to be able to easily monitor the use of TIMP services and respond quickly to problems. A trust system—user interface/user experience (TS-UI/UX) provides a user-friendly visualization interface that can effectively provide information about the trust system to administrators and users.

Storing and managing trust information in the IoT data and social network data collected and received in real time in order to extract and analyze the trust information is very disadvantageous from a cost point of view. A TIMP adopts distributed big data processing clusters using real-time big data processing engines such as Apache Spark, thereby enabling cheap and fast trust analysis.

### 4.2. Trust Data Analytics Procedure

As described in Section 4.1, a trust index is quantitatively or qualitatively calculated and measured based on a trust evaluation model, and then used for the decision-making process not only by value-chains among multiple media stakeholders, but also by applications and service transactions.

The SIoT environment generally consists of IoT devices installed in homes and buildings, network functions for data transmission, IoT platform functions for analyzing data, services/applications using the analyzed information, and the people using them. In this environment, a TIMS should be used to analyze the trust information of users, services, and IoT devices themselves and the trust relationships between them. As mentioned in Section 4.1., a TIMS has various trust analysis functions, such as a social network trust analysis function, a natural language processing trust analysis function, a machine-learning-based trust analysis function, and a semantic-ontology-based trust analysis function. Depending on whether the trustee is a person, a service, or an IoT device, a suitable trust analysis function is selected and used in the TIMS.

For example, a trust analysis via a natural language processing method using text data on a social network service could be used for trust analysis of the user, and a social network analysis function could be used for the trust relationship analysis between the users. Additionally, in order to confirm the trust index of the IoT device itself, a machine-learning-based trust analysis method must be used to determine whether the generated data is in a normal range. The trust relationship analysis of the semantic ontology can be used to identify the trust relationship based on the ownership and usage information between the user and the IoT device.

**Figure 5:**
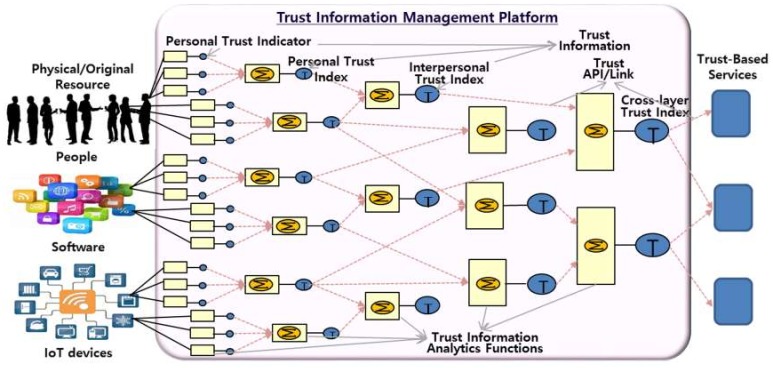
The procedure of trust data analytics.

Thus, in order to analyze trust information between users and devices in a general IoT service such as a smart home, various trust analysis techniques are applied in combination in a TIMS. Here, the trust index between users, the trust index between devices, and the trust index between the device and the user are collected and combined after being individually analyzed. Figure 5 shows a procedural concept in which trust information such as users, devices, and services are collected and combined through subsequent stages to derive a trust index. In most cases, IoT services are a mixture of IoT devices, software, and user-related functions. Therefore, in analyzing trust for these IoT services, it is necessary to derive individual trust indicators and indices for devices, software, and people, as well as cross-layer trust indexes describing from their interactions.

In a TIMS, trust information of a user, a service, or a device, itself derived through individual trust analysis functions such as the natural language processing trust analysis function are structured in RDF format and linked data is stored and managed in the central TripleStore. According to the service requirement, the individual trust information stored and managed in the TripleStore is reconfigured based on the service value chain and transaction relationship, and the trust information is comprehensively calculated.

**Figure 6:**
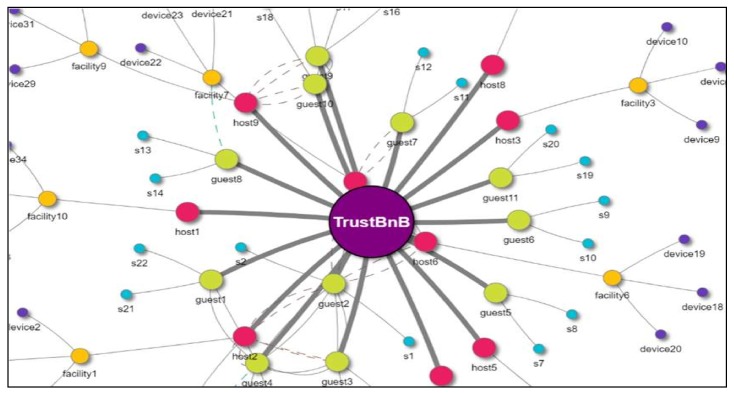
Trust visualization for trust relationship analysis.

In this way, direct and indirect trust relationships can be formed between people, services, and IoT devices. In order to intuitively inform the users of the trust relationship in a variable service environment, a graphic user interface (GUI)-based visualization is effective. Figure 6 shows the trust relationship between the users and the IoT devices owned by a user in the service named TrustBnB. By selecting each path, the trust index between users, services, and IoT devices can be confirmed.

## 5. Implementation and Use Case

In this section, a specific illustration to implement a TIMP is described in detail along with a use case for TIMP-based services.

### 5.1. TIMP Implementation

**Figure 7:**
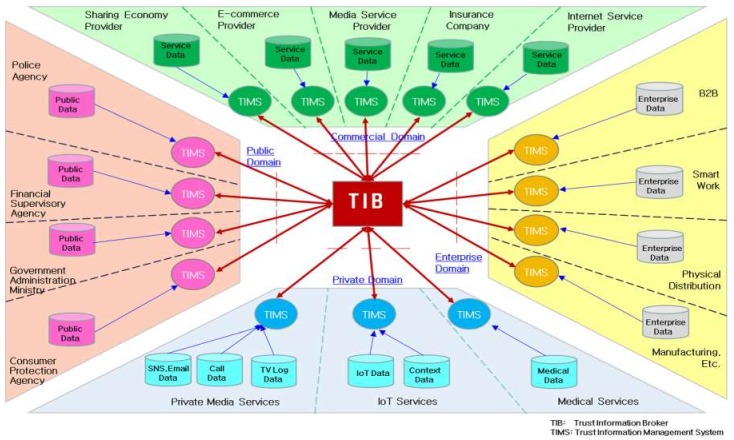
An example of trust information broker implementation.

Figure 7 shows an example of how a TIMS and TIB can be configured and applied to analyze and share trust information in services within the commercial, enterprise, private, and public domains.

The services of each domain should be able to select and configure the TIMS’s functional elements appropriately to the types and attributes of the data they hold and the types and attributes of the trust information they want to receive. The TIMS should be available separately from other service domains and should be able to input and analyze user-, device-, and service-related data held by each service.

In designing and implementing a TIMS to satisfy the needs of each service while reflecting the latest trends in cloud and big data technology, it is cost-effective for a TIMS to use a common service platform based on cloud computing rather than a proprietary system installed in a separate service domain. By adopting a SaaS approach to cloud computing, service providers will be able to access and use trust information services faster and at lower cost by selecting and configuring TAs, TIMS, and TIB capabilities that are appropriate.

**Figure 8:**
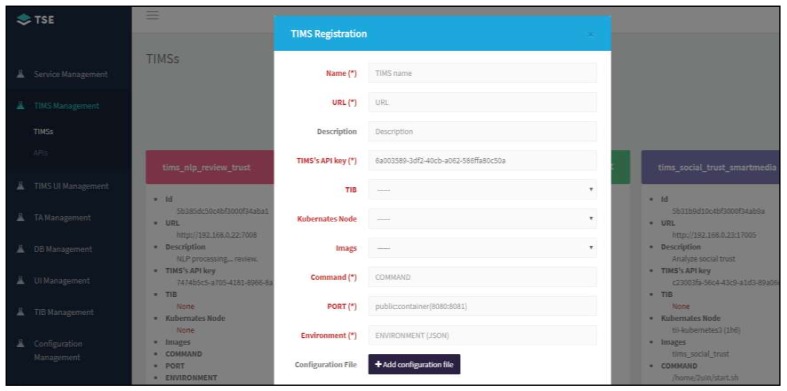
Trust service enabler (TSE) implementation for registering trust systems.

Figure 8 shows a snapshot of a real system implementation for TSE. On the left side, menus for registering TA, database (DB), TIB, and the TIMS constituting the TIMP, as well as menus for orchestrating and connecting them, are shown. The right screen shows examples of the configured trust service using the modules registered in the TSE according to the trust service request, and detailed parameters information such as Id, URL, and the TIMS’s API key for the trust service.

### 5.2. TIMP-Based Service Use Case

As a specific use case with a TIMP, we have illustrated a TIMS-based service, with accommodations/offices and automobiles among the “resources” to which the proposed TIMP is applied.

A resource-sharing service intermediary or broker exists for each field (i.e., accommodations, automobiles, bicycle, facilities, etc.) of a trust-based resource-sharing service. This may be implemented in the form of websites or mobile apps such as Airbnb, Uber, and so on. The resource provider communicates with the website or mobile application of the service intermediary in order to register a shared resource target (accommodations, automobiles, etc.) to provide renting (or sharing), charges, and other required items, and then exchanges information related to the resource-sharing service transaction.

Instead of a lender (or resource provider) or a tenant (or resource user), a service intermediary is responsible for management issues such as use permission limitation of resources such as accommodations/office, automobiles, etc. according to a user’s trust index.

The TIMP can be used for trust-based resource-sharing services during the lease period, using IoT technologies. A TIMP, unlike the existing sharing economy approach (e.g., Airbnb, Uber, and the like) that simply links the owner (or lender) of the resource with the user (or tenant), enables a trustworthy service transaction between a resource provider and a tenant on the basis of the trust information analyzed through accumulated data collected through IoT sensors. The service intermediary may access the user trust information and the resource trust information through the TIMP.

A TIMP-based resource-sharing systems can perform resource-sharing transactions, including the procedures of creating and managing a trust index of a user who uses resources, creating and managing a trust index of resources, and controlling the use of resources based on the user trust index and the resource trust index.

To achieve a trust-based service transaction, the trust information of a user and the trust information of a resource itself is applied based on various technologies, such as IoT, smart home, etc., which is done between a resource provider (e.g., owner, manager, lender, and the like) and a resource user (e.g., tenant, and the like) through a resource-sharing service intermediary that operates a sharing service web page or application system.

The procedure for provision of TIMP-based resource-sharing services is as follows:

- Based on past offer history and reputation or the trust information at the time of resource registration, the resource provider undergoes a verification process for the service target (or shared resource) and the charge.
- A minimum user trust index necessary for use permission of the user when the provider provides a resource is set, and a user trust index set by the provider is compared with the trust index of a user to control the use of the resource in response to the comparison result.
- The TIMP collects resource use status information from the resource and analyzes IoT data from the collected resource to determine whether contract violation, resource failure, or safety problems have occurred; if it is determined that contract violation, resource failure, or safety problems have occurred while the user is using the resource, the provider or the user is notified of this.
- The resource user exchanges information with the service system of the service intermediary; inputs a target resource, a location, a number of users, and the like; searches for an available resource; and exchanges various aspects service transaction information. At this time, the user inputs the required trust level of the resource to be used.
- After the user selects one of the listed resources and makes a reservation, when visiting the accommodation or taking over the automobile at the scheduled time, the user uses the resource according to the contract details.
- The TIMP generates and manages the trust index of a user using the resource by checking IoT data on resource management status (e.g., energy usage, whether a door is locked or not, smoking, etc.) during a resource use period. It can examine whether the service contract has actually been observed through IoT functions (e.g., smoking, whether the number of contracted person is exceeded, safety observance, etc.).
- The TIMP analyzes the trust information as follows:
  . Set-up of functional goals of analysis algorithms;
  . Type of analysis: system trust, personal trust, interpersonal trust (social interaction);
  . Filtering and priority decision based on trust information;
  . Selection of a trust analysis algorithm appropriate for each entity’s type of trust information (e.g., Rule-based algorithm, Machine-based algorithm, Graph-based algorithm, etc.)
- The TIMS calculates a user trust index by adding objective use status data collected from the IoT sensors and subjective data from a resource provider, and by reflecting past history between the user–resource provider entities.
- The TIMP controls the use of the resource based on the user trust index and the resource trust index. It can limit the use of the resource by the user when the comparison result indicates that the trust index of the user to use the resource is lower than the trust index for resource permission set by the provider.
- The TIMP updates the trust information based on the feedback from the user and the resource provider, for example by re-adjusting the trust index of the corresponding user and the trust index of the corresponding resource.

**Figure 9:**
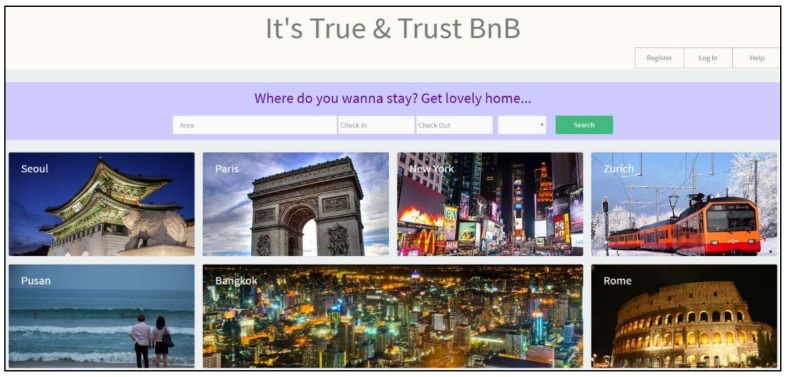
An example of a TIMP-based resource-sharing service (an accommodation service).

Figure 9 shows an accommodations service system depending on a trust-based resource-sharing service. It should be understood that the system structure of Figure 8 organically combines the service intermediary and the TIMP to provide trust-based accommodation renting services between an accommodations provider and a tenant.

**Figure 10:**
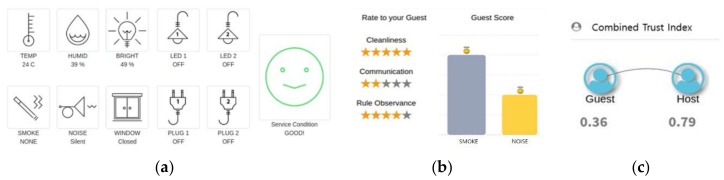
Trust analysis and evaluation at Trust BnB. (**a**) Objective trust analysis with IoT devices; (**b**) subjective trust evaluation; (**c**) trust index.

We conducted trust analysis and evaluation of a Trust BnB service in a sharing guest house, as shown in Figure 10. Figure 10a, below, shows the status of the IoT devices in a Trust BnB service. Through the IoT devices, it is possible to objectively check the trustworthiness of the guest, for example by checking whether the guest has complied with the accommodation contract. Figure 10b represents the host’s subjective evaluation of the guest, and Figure 10c shows the guest’s and host’s trust indexes, which combine objective trust analysis and subjective evaluation.

Although this section used the illustration of accommodations/office resources, the technical scope of the TIMP may be applied to other resources such as bicycles, various facilities, instruments, furniture, and so on.

Trust information in a resource-sharing service can be utilized in terms of each entity, as shown in Table 1:

Rewards such as rate discounts and option changes can provided for future service provision and use through trust information accumulated and updated for users and resources. This allows resource users to use resources cleanly and safely and provide motivation for resource management efforts to resource providers, so that it is possible to enable a trust-based virtuous circle ecosystem. In addition, if necessary, by sharing the trust information of the user, accumulated through the trust-based resource-sharing service, with other services and the third party through the TIB, trust services may be linked and spread.

Note that our implementation and demonstration results based on the international standard ITU-T Y.3052 [11] have been tested and certified from the Telecommunications Technology Association (TTA), Korea, as part of results of the previously conducted Trust Information Infrastructure (TII) project.

## 6. Conclusions

In this paper, we targeted the emerging SIoT environments that will activate the production and distribution of goods and services throughout the ICT industries and the economy by combining the hyper-connectivity provided by IoT and technologies assuring trust in physical things and cyber objects. After designing a trust information management framework, we proposed a TIMP which enables reliable and stable trust-based services by verifying and providing trust information for data, devices, services, and users in SIoT environments where people, objects, and services interact frequently. We implemented core components, including trust data processing and analytics, in the TIMP and demonstrated a use case for TIMP-based services.

Implementing and deploying a TIMP enables the construction of a trustworthy ecosystem while activating SIoT businesses by reducing their transaction costs as well as by eliminating the uncertainties in the use of IoT services and data transactions. In the future, it will be necessary to implement and spread TIMP technologies to all ICT applications and services so that economic ecosystem formation and transaction structures can be dramatically improved.

## Figures and Tables

**Table 1:** Usage of user or resource trust information.

From the Viewpoint of	Usage of User or Resource Trust Information
Resource provider	- Sets the minimum user trust index of a user who is allowed to use the provider’s resource (e.g., select from 1 to 5 stars). - Grant resource use permission only for a trustworthy user. - Suggest differentiated charges and resource use options according to a user trust index (e.g., for five-star graded user, free internet and free parking with a $50 accommodation rental fee; for a three-star graded user, a $60 accommodation rental fee and an extra fee for convenient facilities).
Resource user	- The trust index of a resource is able to be checked through a trust-based resource-sharing service. - Only resources with a trust index are selected by filtering by trust index in a resource use reservation search window. - Resource use with better conditions in the future is possible through observation the resource use rules (or contract) and enhancing user trust.
Resource-sharing service intermediary	- Provision of differentiated resource use fees and options according to trust indexes when the service system is in operation. - The trust-based resource-use service system is configured with a trust index matching method on both sides (e.g., resource provider and user). - In order to increase their user trust index, a user is encouraged to comply with the contract during resource use. - For a provider, a resource trust index is recognized as being linked to increased revenue to encourage efforts to manage users and resources.
